# Effect of a full-process intelligent information management system on the prevention of postoperative infection in patients with indwelling double-J stents after surgery for urinary tract stones: a randomized controlled trial

**DOI:** 10.3389/fsurg.2026.1848686

**Published:** 2026-07-09

**Authors:** Zhihong Huang, Ledan Lin, Lingmin Chen, Hanzhang Huang

**Affiliations:** 1 Nursing Department, Wenzhou People's Hospital, Wenzhou, China; 2 Department of General Surgery, Wenzhou people's hospital, Wenzhou, China

**Keywords:** full-process management, indwelling double-J stent, postoperative infection, preventive effect, urology

## Abstract

**Objective:**

To prospectively compare conventional postoperative management with full-process intelligent information management in patients with indwelling double-J ureteral stents after surgery for urinary tract stones, focusing on delayed stent removal, catheter-related urinary tract infection, discharge teaching quality, social support, and patient satisfaction.

**Methods:**

This was a single-center prospective randomized controlled trial. A total of 100 patients with postoperative indwelling double-J stents after surgery for urinary tract stones who underwent day surgery in the Department of Urology of our hospital between April 2023 and December 2024 were enrolled and randomly assigned, using a random number table, to a control group (*n* = 50) or an intervention group (*n* = 50). The control group received conventional management, whereas the intervention group received full-process intelligent information management in addition to conventional care. The primary outcomes were delayed double-J stent removal and catheter-related urinary tract infection. Secondary outcomes included the Quality of Discharge Teaching Scale (QDTS), the Social Support Rating Scale (SSRS), and patient satisfaction.

**Results:**

Compared with the control group, the intervention group had a significantly lower rate of delayed double-J stent removal (0.00% vs. 10.00%, *P* = 0.048) and catheter-related urinary tract infection (0.00% vs. 12.00%, *P* = 0.023). The intervention group also had a significantly higher QDTS score at discharge (145.32 ± 13.71 vs. 131.54 ± 12.57, *P* < 0.001), a significantly higher post-management SSRS score (45.68 ± 7.82 vs. 37.51 ± 7.89, *P* < 0.001), and a higher overall satisfaction rate (96.00% vs. 82.00%, *P* = 0.025).

**Conclusion:**

Full-process intelligent information management can improve postoperative management in patients with indwelling double-J stents after urinary tract stone surgery, significantly reduce delayed stent removal and catheter-related urinary tract infection, and improve discharge teaching quality, social support, and patient satisfaction.

## 1 Introduction

Urolithiasis is a common urinary system disease worldwide, with an incidence of 6.00%–7.00% in China and a postoperative 5–10-year recurrence rate as high as 50.00% (1). After surgical treatment, a double-J ureteral stent is usually left in place for 1–3 months to maintain urinary tract patency. However, the incidence of double-J stent–related complications is reported to be as high as 80.00% (2). Among these, catheter-related complications，such as urinary tract infection and delayed double-J stent removal (mainly due to forgotten stents), are particularly challenging in clinical practice. Once secondary infection occurs, there is a risk of progression to urosepsis, and delayed stent removal can result in stone formation, greatly increasing patient suffering and medical costs (3).

Conventional management mostly relies on oral education and telephone follow-up, which is essentially a “passive reminder” model lacking the capability for direct intervention in complications. Previous studies have mainly focused on optimizing management processes and have not integrated pharmacological prophylaxis into a closed-loop system of “systematic management combined with pharmacologic protection” (4). Qiu et al. (5) demonstrated that full-process intelligent information management system can significantly improve the quality of nursing care in wards and help prevent adverse events. In this study, we have established a full process management refers to a structured management strategy that covers the entire care pathway from perioperative assessment to post-discharge follow-up and timely stent removal. It comprised five core components: (1) establishment of a multidisciplinary urology medical-nursing team; (2) an intelligent reminder and follow-up system; (3) perioperative infection-prevention measures; (4) multimodal health education; and (5) checklist-based continuity of care. And collaborate with the hospital Information system(HIS) to register information related to double-J tubes, record planned extubation dates, issue reminders to clinical doctors, and link with patient follow-up reminders.

Therefore, the aim of this study was to prospectively compare conventional management with full-process intelligent information management in patients with postoperative indwelling double-J stents after surgery for urinary tract stones. Specifically, we compared delayed stent removal, catheter-related urinary tract infection, discharge teaching quality, social support, and patient satisfaction between the two groups.

## 2 Materials and methods

### 2.1 General data

This study was a single-center, prospective, randomized controlled trial conducted in the Department of Urology of Wenzhou People's Hospital. A total of 100 patients who underwent surgery for urinary tract stones and received postoperative indwelling double-J stents were enrolled consecutively from April 2023 to December 2024 and randomly assigned in a 1:1 ratio to the control group or the intervention group using a random number table.

**Inclusion criteria:**

1. Patients who met the surgical indications for urinary tract stones and successfully underwent postoperative indwelling of a double-J stent;
2. No evidence of urinary tract infection before surgery;
3. Able to communicate effectively and complete follow-up procedures.

**Exclusion criteria:**

1. Bladder dysfunction such as adenomatous or interstitial cystitis and urethral syndrome, or other bladder, renal, urethral, or ureteral diseases;
2. Hematopoietic or coagulation disorders, or emotional/behavioral abnormalities;
3. Severe infection, renal atrophy, or pregnancy;
4. Indwelling double-J stent due to urinary tract tumors or ureteral fibrosis;
5. Very poor compliance or coexisting psychiatric disorders.

**Withdrawal criteria:** Deterioration of condition or voluntary withdrawal after enrollment.

Patients were randomly assigned to a control group (*n* = 50) and an intervention group (*n* = 50) using a random number table.

Control group (*n* = 50): 31 males and 19 females; age 18–75 years (51.42 ± 5.89); body mass index (BMI) 18–30 kg/m^2^ (23.54 ± 5.64).Intervention group (*n* = 50): 32 males and 18 females; age 18–75 years (51.57 ± 6.34); BMI 18–30 kg/m^2^ (23.54 ± 5.64).There were no significant differences in baseline characteristics such as age, sex, and BMI between the two groups (*P*＞0.05), indicating comparability.

This study complied with the principles of the Declaration of Helsinki (6) and was approved by the Ethics Committee of Wenzhou People's Hospital (Approval No. KY-2022-085). All enrolled patients were fully informed about the study and signed written informed consent. The study involved optimization of clinical nursing and management models, and the intelligent information system consisted of integrated and optimized modules based on existing hospital functions.

### 2.2 Surgical procedures

All patients received double-J ureteral stents of 4.7F,5F and 6F, selected according to the patient's anatomy and clinical condition. The surgical procedures included ureteroscopic lithotripsy, flexible ureteroscopy, or percutaneous nephrolithotomy.

## 3 Methods

### 3.1 Control group

The control group received routine care plus telephone follow-up, including health education, dietary guidance, and reminders of stent removal time. In terms of activity, patients were advised to avoid vigorous bending, prolonged sitting, and other movements that might cause traction on or displace the double-J stent. A low-oxalate diet was recommended, and patients were instructed to maintain a daily fluid intake of 2,000–3,000 mL to promote urine excretion. The clinical significance of stent placement (e.g., maintaining ureteral patency and preventing postoperative obstruction) and the risks of overdue removal (such as peri-stent stone formation or encrustation) were explained in detail. Based on the surgical procedure, patients were clearly informed of the scheduled stent removal time, usually 1–2 weeks postoperatively.

Telephone follow-up was conducted at standardized time points, with one follow-up at 1 week after surgery. The content included inquiries about discomfort symptoms such as flank pain, urinary frequency, and gross hematuria; reminders to complete a routine urine test before stent removal; and assistance with scheduling outpatient appointments for stent removal to ensure timely return.

### 3.2 Intervention group

On the basis of conventional care provided to the control group, the intervention group received additional full-process management, as follows:

#### 3.2.1 Establishment of a multidisciplinary team and clarification of responsibilities

A full-process medical–nursing management research team was established, including two attending urologists, one chief urologist, five primary nurses, and two specialist nurses from the cystoscopy unit, forming a “seamlessly linked” working model. All team members underwent training according to standardized procedures. Attending physicians were responsible for outpatient education, development of individualized treatment plans, preoperative risk communication, scheduling of postoperative follow up, and perioperative management of day surgery patients. Primary nurses were responsible for education throughout the entire process, covering perioperative health guidance and post-discharge continuity of care, including dietary precautions, drug dosage and timing, and suitable exercise patterns. Nurses in the cystoscopy unit focused on perioperative education for day surgery patients, with emphasis on preparation before cystoscopic stent removal.

#### 3.2.2 Construction of an intelligent information system

1. To reduce the risk of delayed stent removal, a three-level intelligent reminder mechanism was established.
  **Intelligent stent removal reminder:** During hospitalization, the bedside Ya-Hua display system showed in real time the stent location, model, and scheduled removal time. After discharge, a cloud follow-up system provided three-level reminders: 7 days before removal, the hospital HIS system alerted the attending physician; 3 days before removal, a text message reminder was sent to the patient; and 1 day before removal, nurses conducted a telephone confirmation. This multi-dimensional strategy targeted the problem of delayed stent removal.
  **App-based medication reminder:** Through a post-treatment follow-up app linked to the hospital system, daily medication reminders were set to push information on the timing and dosage of drugs such as levofloxacin. Patient confirmation was recorded to improve adherence.
  **Urine test reminder:** The system automatically sent SMS or app reminders 7 days before stent removal, prompting patients to complete a routine urine examination to obtain results before removal, thereby supporting infection risk assessment and reducing the likelihood of forgotten stent removal.
2. To achieve early infection warning, an AI risk prediction module was integrated. The model incorporated variables such as body temperature, urinary leukocyte count, and patient-reported “bladder irritation” symptoms (e.g., urinary frequency and urgency), and calculated a risk value using a logistic regression model. When the risk value exceeded 0.6, an automatic alert was sent to medical and nursing staff, enabling early identification of infection.
3. To facilitate early intervention for urinary tract infection, an IoT urine flow monitoring module was added. After discharge, patients wore a portable uroflow sensor that continuously monitored urine flow rate (normal reference value ＞15 mL/s; ＜10 mL/s suggests catheter obstruction), allowing intervention 2–3 days earlier compared with conventional “symptom-driven” management.

#### 3.2.3 Perioperative infection prevention

Efforts were made to shorten stent dwelling time as much as possible or replace the stent promptly, and to increase urine output. Urinary tract infection was controlled, and citrate therapy was applied as appropriate. Anti-encrustation or coated stents were used to reduce stone formation on the stent surface.

Perioperative infection prevention in the intervention group did not mean indiscriminate acceleration of stent removal for all patients. Instead, the planned stent removal date was determined according to the surgical procedure and postoperative recovery, usually within 1–2 weeks in this day-surgery pathway. The full-process management team attempted to achieve the earliest clinically appropriate removal within the planned window. Stent replacement was performed only when continued drainage was clinically necessary, such as persistent ureteral edema or obstruction, residual stones requiring staged treatment, or suspected stent dysfunction. These events should be reported explicitly in the Results section if they occurred.

#### 3.2.4 Multimodal health education

Multimodal education was implemented, including development of standardized oral education templates for the preoperative, postoperative, and post-discharge stages; production of educational materials and brochures with graphical illustrations of complication recognition; and creation of a 5–8 min “double-J stent care practice video” showing correct hydration and activity methods. A 1:1 anatomical model of the double-J stent was used to visually demonstrate stent position, and nurses provided hands-on demonstration of perineal hygiene to reduce infection risk.

Through the hospital's WeChat public account, a pre-admission education program—“Preoperative Instructions for Double-J Stent Placement”—was delivered, along with a day-surgery pathway covering preoperative assessment, intraoperative cooperation, and 2-hour postoperative observation. A shared decision-making model was adopted: medical and nursing staff jointly assessed patients' understanding of stent management and decision-making needs, provided 2–3 stent removal time options with explanation of pros and cons, and guided patients to select a plan that accommodated their work schedule. One week after surgery, satisfaction with the decision-making process was assessed using a 5-point Likert scale.

#### 3.2.5 Checklist-based follow-up and innovative continuity of care

Follow-up forms were customized according to stone location and postoperative recovery level, including 10 indicators such as stent-related symptoms, daily fluid intake, and review plans, and were embedded into the cloud follow-up system for online completion by medical staff. This allowed dynamic assessment of post-discharge stent status.

A multi-dimensional continuity-of-care network was constructed. At discharge, nurses recorded the discharge diagnosis and planned stent removal date on a follow-up contact card, and patients could present the card at any of the three community hospitals within the hospital's medical consortium to complete follow-up and stent removal. Using a 5G internet hospital platform combined with augmented reality (AR) technology, remote guidance was provided to help patients identify abnormal urine color. One urologist was assigned weekly to provide outpatient services at consortium institutions, and a 24 hour home-care hotline was opened to answer daily questions.

A “Post-Discharge Care Questionnaire for Patients with Indwelling Double-J Stents” was administered at discharge and 1 month after discharge to assess adherence to hydration (≥2,000 mL/day considered adequate), compliance with activity recommendations, and other aspects. For identified problems, on-site guidance was provided to reinforce key points of stent care, including avoidance of urinary retention and regular urine testing.

### 3.3 Outcome measures

#### 3.3.1 Delayed double-J stent removal rate

In this study, postoperative double-J stent removal was routinely scheduled within 1–2 weeks after surgery according to the surgical procedure and the treating urologist's assessment. Failure to remove the stent within 14 days after surgery was defined as delayed double-J stent removal, unless prolonged indwelling or planned replacement was clinically justified and documented in the medical record.

#### 3.3.2 Quality of discharge teaching

The QDTS was used to evaluate satisfaction, comprehension, and quality of discharge guidance. The scale comprises two dimensions: discharge teaching content (6 items) and discharge teaching skills (12 items), each item scored 0–10 points. The total score is the sum of the two dimensions; higher scores indicate better quality of discharge teaching.

#### 3.3.3 Social support

The SSRS was used to evaluate support during treatment and rehabilitation. It includes subjective support (4 items), objective support (3 items), and utilization of support (3 items), with a total score ranging from 12 to 66 points. Higher total scores indicate better social support.

#### 3.3.4 Complication rate

The number of complications divided by the number of indwelling double-J stents during the same period, multiplied by 100.00%.

#### 3.3.5 Satisfaction

Satisfaction (%) = (Actual score/total score)×100.00%

Satisfaction levels: very satisfied, 100 points; satisfied, 80 points; fair, 60 points; dissatisfied, 40 points; very dissatisfied, 20 points.

### 3.4 Statistical analysis

This study was designed as an exploratory prospective randomized controlled trial. At the time of protocol development, robust preliminary data for a formal sample size calculation were not available. Therefore, the sample size was determined pragmatically according to the annual case volume of the day-surgery unit, study feasibility, and the need to obtain preliminary effect estimates for a future confirmatory trial.

An excel database was established, and baseline characteristics and study data of the control group (*n* = 50) and intervention group (*n* = 50) were coded, classified, and then analyzed using SPSS 23.0 software. Continuous variables were first assessed for distributional normality. Normally distributed variables were expressed as mean ± standard deviation and compared using the independent-samples t test; non-normally distributed variables should be compared using the Mann–Whitney U test. Categorical variables were expressed as *n* (%) and compared using the chi-square test or Fisher's exact test, as appropriate. All tests were two-sided, and *P* < 0.05 was considered statistically significant.

## 4 Results

Compared with the control group, the intervention group showed better outcomes in all predefined comparison domains. Specifically, the intervention group had a higher QDTS score at discharge, a higher post-management SSRS score, a lower rate of delayed double-J stent removal, a lower rate of catheter-related urinary tract infection, and a higher overall satisfaction rate.

### 4.1 Comparison of adverse reaction rates between the two groups

As shown in Table 1, the delayed double-J stent removal rate and catheter-related urinary tract infection rate in the intervention group were lower than those in the control group (*P*＜0.05).

**Table 1:** Comparison of adverse reaction rates between the two groups [*n* (%)].

Group	*n*	Delayed double-J stent removal *n* (%)	Catheter-related urinary tract infection *n* (%)
Control	50	5 (10.00)	6 (12.00)
Intervention	50	0 (0.00)	0 (0.00)
*χ^2^*	–	3.951	5.144
*P*	–	0.048	0.023

### 4.2 Comparison of QDTS and SSRS scores between the two groups

As shown in Table 2, there was no significant difference in pre-management SSRS scores between the two groups (*P* ＞ 0.05). After management, the total SSRS score in the intervention group was significantly higher than that in the control group (*P* ＜ 0.05). At discharge, the total QDTS score in the intervention group was also higher than that in the control group.

**Table 2:** Comparison of QDTS scores at discharge and SSRS scores before and after management between the two groups.

Group	*n*	QDTS score at discharge (points)	SSRS score (points) pre-management	SSRS score (points) post-management
Control	50	131.54 ± 12.57	25.61 ± 8.57	37.51 ± 7.89
Intervention	50	145.32 ± 13.71	25.67 ± 7.53	45.68 ± 7.82
*t*	–	5.239	0.037	5.201
*P*	–	＜0.001	0.970	＜0.001

### 4.3 Comparison of management satisfaction between the two groups

As shown in Table 3, the overall satisfaction rate in the intervention group (96.00%) was higher than that in the control group (82.00%) (*χ^2^* = 5.005, *P* = 0.025, *P* ＜ 0.05).

**Table 3:** Comparison of management satisfaction between the two groups.

Group	*n*	Very satisfied	Satisfied	Dissatisfied	Overall satisfaction *n* (%)
Control	50	26	15	9	41 (82.00)
Intervention	50	34	14	2	48 (96.00)
*χ^2^*	–	–	–	–	5.005
*P*	–	–	–	–	0.025

## 5 Discussion

The duration of double-J stent indwelling correlates with disease severity in patients undergoing surgical treatment for urinary tract stones: the more severe the condition, the longer the stent is likely to remain in place. Among perioperative complications related to double-J stents, infection is particularly troublesome. It has been reported that approximately 42%–100% of ureteral stents become colonized by bacteria, which may induce or exacerbate lower urinary tract symptoms (6, 7). Early recognition of postoperative infection in patients with indwelling stents is challenging, often because of nonspecific early symptoms and the complexity of pathogen identification (8, 9). If inadequately managed, infection may progress, leading to complications such as urosepsis and peri-stent stone formation or encrustation (10–12), thereby posing a significant threat to patients' health and prognosis. Studies have shown that when the stent dwell time exceeds 30 days, the risk of urosepsis increases five-fold. Early removal of ureteral stents can effectively reduce the incidence of stent-related complications (7, 13). Therefore, on the basis of a thorough understanding of indications for double-J stent use, preventing postoperative infection related to stent indwelling not only alleviates patients' physical and psychological burden, but also reduces treatment costs and improves overall medical quality.

In this study, the lower urinary tract infection rate observed in the intervention group may be attributed to early warning by the AI system and the timely interventions triggered thereby (e.g., guidance on repeat urine testing, adjustment of fluid intake, or prompt medical consultation), along with standardized perioperative antibiotic prophylaxis.

Currently, in other countries, management of patients with indwelling double-J stents mainly depends on community-based care and continuity of care; however, postoperative infection and delayed stent removal still occur. Ziemba et al. (14) confirmed that a ureteral stent tracker (UST) can reduce the likelihood of forgotten stent removal. Huang H et al. (15) showed that standardized continuity of care for patients with indwelling double-J stents can reduce the incidence of complications. In our study, the delayed stent removal rate in the intervention group was 0%, significantly lower than that in the control group. This can be directly attributed to the intelligent reminder system, which provided multiple safety layers through the hospital HIS, patient app, and nurse telephone calls, effectively preventing the major risk of “forgotten stent removal.”

This study also adopted patient-reported outcome measures, such as satisfaction and QDTS scores. Although these indicators are somewhat subjective, they reflect patients' perceived experience of medical services and internalization of health education, and therefore represent an important dimension of healthcare quality. The significant improvements in these indicators in the intervention group suggest that full-process management can enhance doctor–patient communication, deliver continuous informational support, and provide personalized follow-up care, thereby improving patients' subjective experiences and self-management ability. Future research could further strengthen the robustness of these conclusions by incorporating more objective biological indicators, such as inflammatory marker levels, and by using blinded assessment where feasible.

However, This management strategy depends partly on patients'ability to use mobile communication tools, understand reminders, and cooperate with follow-up. Therefore, its applicability may be limited in patients with poor digital literacy, communication barriers, or inadequate family support. For such patients, simplified reminder pathways, caregiver-assisted management, or more intensive manual follow-up may be necessary.

In summary, full-process intelligent information management, consisting of a multidisciplinary team, HIS-based intelligent reminders, perioperative infection-prevention measures, multimodal education, and checklist-based continuity of care, was associated with lower rates of delayed stent removal and catheter-related urinary tract infection, as well as better discharge teaching quality, social support, and patient satisfaction in patients with postoperative indwelling double-J stents after urinary tract stone surgery.

## Data Availability

The raw data supporting the conclusions of this article will be made available by the authors, without undue reservation.
